# Identification of Important Genes of Keratoconus and Construction of the Diagnostic Model

**DOI:** 10.1155/2022/5878460

**Published:** 2022-09-12

**Authors:** Lin Wang, Yuqing Wang, Juan Liu, Wencheng Zhao

**Affiliations:** ^1^Eye Hospital, The First Aﬃliated Hospital of Harbin Medical University, Harbin, China; ^2^Ningde Municipal Hospital Affiliated with Ningde Normal University, Fujian, China; ^3^The First Affiliated Hospital of Harbin Medical University, Harbin, China

## Abstract

**Objective:**

The aim of the study is to investigate the potential role of keratoconus (KC) in the diagnosis of keratoconus (KC).

**Methods:**

GSE151631 and GSE77938 were downloaded from the comprehensive gene expression database (GEO). By using the random forest model (RF), support vector machine model (SVM), and generalized linear model (GLM), important immune-related genes were identified as biomarkers for KC diagnosis.

**Results:**

Through the LASSO, RFE, and RF algorithms and comparing the three sets of DEGs, a total of 8 overlapping DEGs were obtained. We took 8 DEGs as the final optimal combination of DEGs: AREG, BBC3, DUSP2, map3k8, Smad7, CDKN1A, JUN, and LIF.

**Conclusion:**

Abnormal cell proliferation, apoptosis, and autophagy defects are related to KC, which may be the etiology and potential target of KC.

## 1. Introduction

Keratoconus (KC) is a bilateral progressive, noninflammatory corneal stromal thinning disease. It is characterized by corneal conical dilation and a series of corneal curvature changes. The suspicious factors related to its occurrence include genetic mechanisms, family-related, allergic diseases, and specific diseases [1]. In the early stage of onset, it is mainly manifested in the continuous growth of myopia and astigmatism, which seriously affects the vision of patients. If it continues to develop, the final result is corneal transplantation [2]. The etiology of KC is not clear. So, exploring its etiology and pathogenesis and taking effective control measures are the fundamental to prevent and treat keratoconus. With the technological development of molecular biology, research on KC has gone deep into the molecular level. The cornea is located in the front of the eye, and its anatomical structure is relatively clear, which is convenient for technical operation and observation of the gene transfection process. At the same time, the particularity of corneal immune amnesty makes it an ideal target organ for molecular therapy [3].

In this paper, multiple KC-related high-throughput detection data are collected for analysis, and the KC significantly related genes are compared and screened. The important genes significantly related to immunity are screened by using the sample immune evaluation and WGCNA algorithms, and then, the internal correlation of the pathway is analyzed by combining the KEGG cross talk between the important genes. Finally, different optimization algorithms are used to further screen the characteristic genes, and the sample diagnosis classification model is constructed. The purpose of this study was to explore the role of immune-related genes in KC by comparing the complete gene expression profile data downloaded from the gene expression comprehensive database (GEO), and we further try to use immune-related genes as diagnostic biomarkers of KC patients, which may be helpful to the diagnosis and treatment of KC. In addition, we also studied the potential relationship between immune cells and KC.

## 2. Methods

### 2.1. Data Search and Information

In NCBI GEO (https://www.ncbi.nlm.nih.gov/GEO/), we search the database with “keratoconus” as the keyword, and the data filtering criteria are as follows: (1) transcriptome expression profile data of human corneal tissue samples; (2) classification of patients with KC and Ctrl control samples; (3) the number of samples available for inclusion in the analysis shall not be less than 20. Three sets of datasets meeting the requirements (all high-throughput detection expression profile data) were obtained. The information is shown in Table 1. We combined the first two datasets (GSE151631 and GSE77938) in Table 1 as the main analysis dataset of this analysis and remaining GSE112155 as the verification dataset of this analysis.

### 2.2. Screening of Significantly Differentially Expressed Genes

We removed the batch effect, combined the samples, and screened the significantly differentially expressed genes. We removed the batch effect, combined the samples, and screened the significantly differentially expressed genes becauseof the level of gene expression in batch is different from that in r3.0 6.1 SVA in language Package (https://www.bioconductor.org/packages/release/bioc/html/sva.html). Version 3.38.0 removed the batch effect of GSE151631 and GSE77938 datasets and then obtained the combined expression level data.

In the combined expression profile dataset, we used R3 6.1 limma package version 3.36.0 in language (https://www.bioconductor.org/packages/release/bioc/html/edgeR.html). The differentially expressed genes (DEGs) between KC vs. Ctrl groups were screened, and FDR < 0.05 and |log2fc| > 1 were selected as the threshold for screening DEGs. Then, based on the DEGs obtained by screening, the R3 Pheatmap package in 6.1 language (version 1.0.8) was used (https://cran.r-project.org/package=pheatmap). Bi-directional hierarchical clustering based on Euclidean distance is performed on the expression values and displayed with a heat map.

### 2.3. Screening of DEGs Significantly Related to Important Immune Cells

We evaluated the sample immune cell types based on the ssGSEa algorithm. The concept of a disease immune microenvironment means that a large number of immune cells are often gathered inside and around the disease. There are countless links between these immune cells, as well as between tumor cells and immune cells. There are a variety of immune cells, so the so-called immune microenvironment, or immune infiltration analysis, is essential to find out the composition proportion of immune cells in the tissue. This paper adopts R3 based on ssGSEa (single sample gene set enrichment analysis) algorithm 6.1 GSVA (https://www.bioconductor.org/packages/release/bioc/html/GSVA.html). We used version 1.36.3 (gene set variation analysis for microarray and RNA SEQ data) to evaluate the immune characteristics of each sample and then used the inter-group *t*-test to analyze the differences in the distribution of various types of immune cells between KC and Ctrl groups and retained the immune cell types with significant differences in the distribution between groups.

We screened DEGs that are significantly related to important immune cells. Then, the expression level of DEGs screened in the second step and the immune cell types with significant differences between KC vs. Ctrl groups evaluated by ssGSEa were analyzed by R3 6.1 cor function in language (https://77.66.12.57/R-help/cor.test.html). The Pearson correlation coefficient (PCC) between them was calculated. The DEGs with significance *p* value less than 0.05 and absolute PCC values higher than 0.5 were retained as DEGs significantly related to important immune cells.

### 2.4. WGCNA Algorithm Screens Modules Significantly Related to Disease Status and Immunity

Weighed gene coexpression network analysis (WGCNA) is a bioinformatics algorithm for constructing the coexpression network, which is used to identify modules associated with diseases and screen important pathogenic mechanisms or potential therapeutic targets. Based on the expression levels of all genes in the combined samples, we used R3 6.1 WGCNA package version 1.61 in language (https://cran.r-project.org/web/packages/WGCNA/index.html). We screen modules significantly related to the disease status and immune cells of the sample. The screening threshold of module division is as follows: the module set contains at least 100 genes, cutheight = 0.995.

Then, the DEG set significantly related to important immune cells screened in the third step is mapped to each WGCNA module, and the significant enrichment parameter fold enrichment and the enrichment significant *p* value in the module are calculated by Fisher's exact test. The selection of the module screening threshold *p* < 0.05 and fold enrichment is >1. The genes significantly enriched in the module are taken as the object of further analysis and research.

### 2.5. Construction of the KEGG Cross talk Network Related to Important Genes

We used David6 8 (https://david.ncifcrf.gov/) online analysis tools (https://david.ncifcrf.gov/). The important immune-related genes obtained in the fourth step were enriched in the module by KEGG signal pathway enrichment analysis (FDR <0.05). Then, we used R3 6.1 GSVA version 1.36.3 in language [4] Based on the expression level of genome-wide genes in the combined samples, the KEGG signal pathway related to significant enrichment was quantitatively analyzed, and R3 cor function in 6.1 language calculates the correlation PCC between the quantified KEGG signal pathways, retains the part with the correlation significance *p* value less than 0.05 and the correlation absolute value higher than 0.5, and finally constructs the pathway cross talk network through Cytoscape version 3.9.0 (https://www.cytoscape.org/). The genes involved in the KEGG cross talk network were further analyzed as important genes.

### 2.6. Optimization of Immune-Related Marker Screening and Construction of the Diagnostic Model

We used the optimization algorithm to screen important immune-related markers. Taking the combined dataset samples as the training set, three different optimization algorithms are used for gene feature screening: LASSO (least absolute shrinkage and selection operator), RFE (recursive feature extraction), and RF (random forest). (1) We adopt Lars package version 1.2 in the R 3.6.1 language (https://cran.r-project.org/web/packages/lars/index.html). The target gene set was analyzed by regression to screen the characteristic genes; (2) we use R3 6.1 caret package in the language (https://cran.r-project.org/web/packages/caret) (version 6.0–76) RFE algorithm to screen for the optimal combination of characteristic immune-related DEGs; we use R3 randomForest package version 4.6–14 of 6.1 (https://cran.r-project.org/web/packages/randomForest/). The bootstrap algorithm is used to screen the optimal combination of characteristic immune-related DEGs. Then, we compare the results of the three algorithms and select the overlapping part as the final combination of feature immune-related DEGs.

### 2.7. Construction and Verification of the SVM Diagnostic Model

We adopted SVM (support vector machine, SVM) methods of R3.6.1 e1071 version 1.6–8 (https://cran.r-project.org/web/packages/e1071) and constructed an SVM classifier based on optimal feature gene combination (Core: sigmoid kernel; cross: 100 fold cross-validation). SVM is a supervised classification algorithm of machine learning. Through the eigenvalues of features in each sample, it can distinguish and predict the samples and estimate the probability that they belong to a certain category so as to realize the discrimination of sample types.

Then, based on the selected characteristic immune DEG factors, we used R3 6.1 language rmda package version 1.6 (https://cran.r-project.org/web/packages/rmda/index.html). We analyze the decision curve of the single DEG and multiDEG combined models, respectively and observe the net return of each DEG factor on survival and prognosis results so as to compare the effects of different factors on sample types.

In addition, we also use R3.4.1 proc (https://cran.r-project.org/web/packages/pROC/index.html). Version 1.12.1 package evaluates the constructed SVM classification model and calculates various index values of the ROC curve: area under the curve (AUROC), sensitivity (SEN), and specificity (SPE). The ROC curve is one of the main evaluation indexes used by classification models, especially binary classification models. Its working principle is to give a model, input a set of data of known positive and negative classes, and measure the performance of the model by comparing the prediction of the model to this set of data. AUROC is the quantitative index of the ROC curve, and its value is distributed between 0.5 and 1. The closer it is to 1, the better the classifier performance. Finally, in the validation dataset GSE112155, the effectiveness of the diagnostic model is verified.

### 2.8. Expression Level of Characteristic Immune DEGs and Correlation Analysis with Related Immune Cells

We used R3 6.1 the cor function in the language and calculated the PCC between the expression level of characteristic immune DEGs screened and the related immune cell types with significant differences in distribution among groups screened in the third step and then, we display the correlation to observe the correlation between the expression level of characteristic DEGs and related immune cells.

### 2.9. Screening of Small Molecules of Chemical Drugs Related to Characteristic Immune DEGs

From updated comparative toxicology database 2021 (https://ctd.mdibl.org/), we download all gene chemical connections and then search for small chemical molecules directly related to KC disease in the CTD database with “keratoconus” as the keyword. First, we extract the small chemical molecules connected with characteristic immune DEGs from all gene chemical connections and then select the small chemical molecules directly related to KC disease from these small molecules so as to obtain the small chemical molecules of KC disease associated with characteristic immune genes.

## 3. Results

### 3.1. Screening of Significantly Differentially Expressed Genes

**Figure 1:**
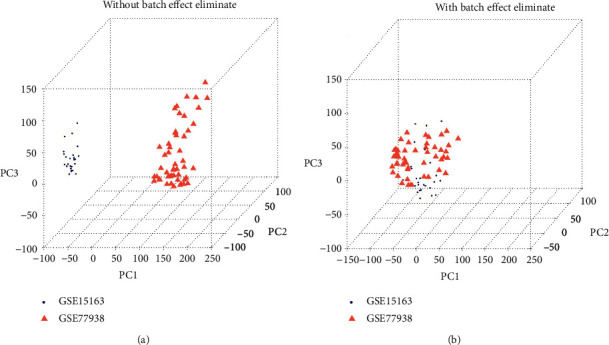
(a) Without batch effect eliminate. (b) With batch effect eliminate.

We download the corresponding expression profile data (see Table 1 for the expression profile data of each dataset before merging, and the data format is the gene read count expression level after standardization under the detection platform of each dataset), as described in the method. As described in the method, first, we remove the batch effect of the two datasets (GSE151631 and GSE77938) through the SVA algorithm and then merge them into one dataset. The sample relationship before and after batch effect removal is shown in Figure 1. It can be seen from the figure that the two datasets before batch effect removal are arrays, which are obviously distributed in different regions. After batch effect removal, the two datasets tend to be distributed together. The expression spectrum data of the combined datasets are shown in Table 2.

**Figure 2:**
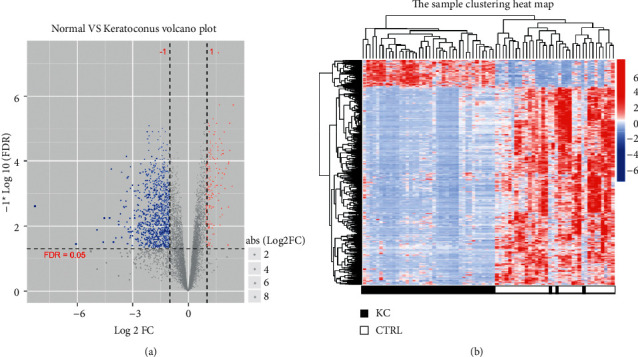
(a) Normal vs keratoconus volcano plot. (b) The sample clustering heat map.

Then, the edger algorithm is used to analyze and screen the DEGs of the KC (44 samples) vs. Ctrl (32 samples) groups in the combined dataset, and a total of 1246 DEGs meeting the threshold conditions are obtained. The test volcanic diagram is shown in Figure 2(a), and the list of DEGs is shown in Table 3. The sample clustering heat map is made based on the DEG expression level obtained by screening, as shown in Figure 2(b). It can be seen from the cluster diagram that different types of samples can be separated based on the DEG expression value obtained by screening, and the color is clear, indicating that the DEGs obtained by screening have expression characteristics.

### 3.2. Screening of DEGs Significantly Related to Important Immune Cells

**Figure 3:**
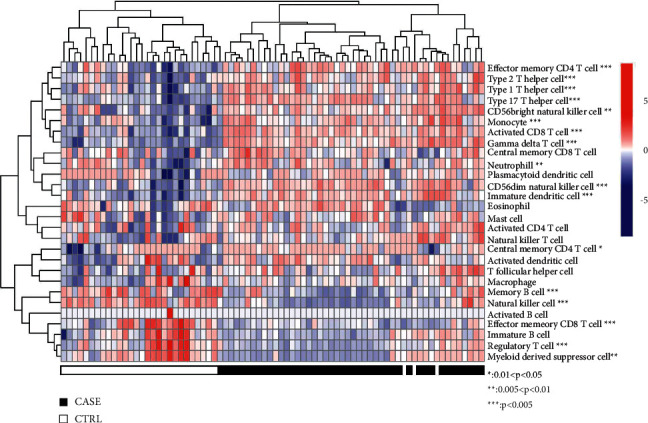
Based on the gene expression profile data detected in the combined samples, the immune fine of each sample is calculated by ssGSEa algorithm. According to the division of cell types, the proportion of 28 immune cell types is obtained. The display diagram is shown in Figure 3.

Based on the gene expression profile data detected in the combined samples, the immune fine of each sample is calculated by the ssGSEa algorithm. According to the division of cell types, the proportion of 28 immune cell types is obtained. The display diagram is shown in Figure 3. Then, we compare the proportion of various immune cells in kcvs. According to the differences between Ctrl groups, 18 kinds of immune cells with significant differences were obtained: gamma delta T cell, type 17 T helper cell, CD56dim natural killer cell, monocyte, natural killer cell, activated CD8 T cell, image dendritic cell, effector memory CD4 T cell, type 1 T helper cell, memory B cell, effector memory CD8 T cell, regulatory T cell type 2 T helper cell, CD56bright natural killer cell, myeloid-derived suppressor cell, neutrophil, eosinophil, and central memory CD4 T cell. Then, the expression level of DEGs was screened in Step 1, and 18 immune cell types with significant differences between KC vs. Ctrl groups evaluated by ssGSEa were analyzed by R3 6.1; the cor function in the language calculates the PCC between them. Only the DEGs with a correlation significance *p* value less than 0.05 and a PCC absolute value higher than 0.5 are retained as the DEGs significantly related to important immune cells. A total of 6716 pairs of correlation action pairs were obtained and involved 1046 DEGs.

### 3.3. WGCNA Algorithm for Screening Disease Progression and Immune-Related Modules

**Figure 4:**
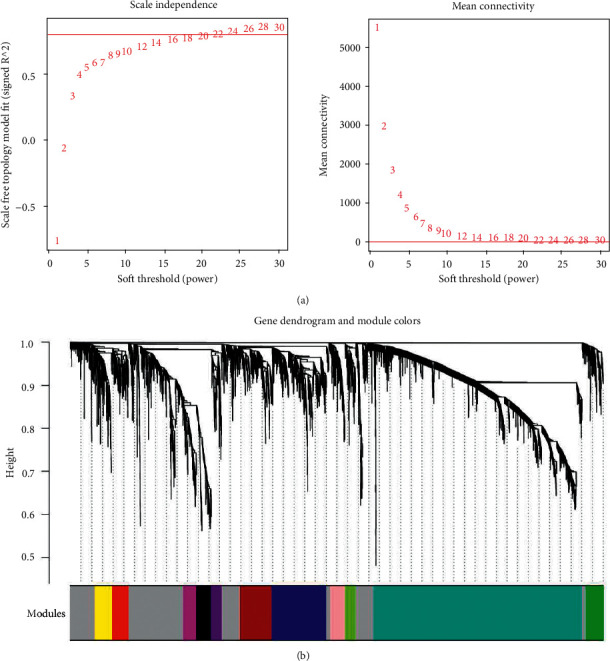
As shown in Figure 4(a), we select the value of power when the square value of correlation coefficient reaches 0.9 for the first time, that is, power = 22. As shown in Figure 4(b), a total of 12 modules are obtained.

**Figure 5:**
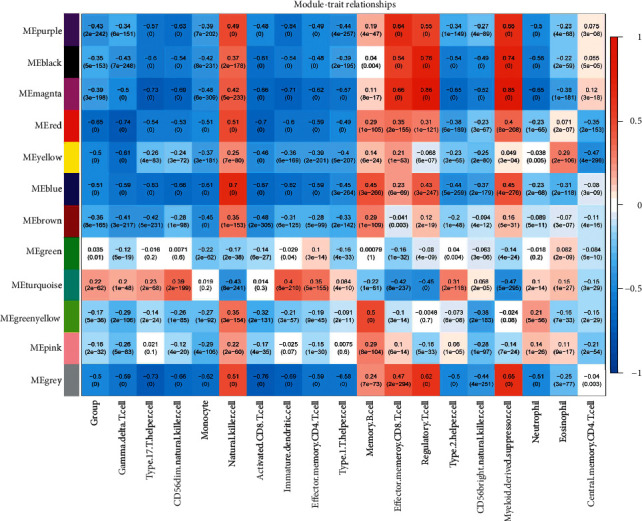
Finally, calculate the correlation between the 18 important immune information screened in the second step and each module divided, as shown in Figure 5.

The expression levels of all genes detected in the combined samples were analyzed. In order to meet the preconditions of scale-free network distribution as much as possible, we need to explore the value of the weight parameter power of the adjacency matrix. We set the selection range of network construction parameters and calculate the scale-free distribution topology matrix. As shown in Figure 4(a), we select the value of power when the square value of the correlation coefficient reaches 0.9 for the first time, that is, power = 22. Then, the average node connectivity of the constructed coexpression network is 1, which fully conforms to the nature of a small-world network. Then, we calculate the dissimilarity coefficient between nodes and obtain the systematic clustering tree. We set the minimum number of genes in each module as 100 and the pruning height as cutheight = 0.995. As shown in Figure 4(b), a total of 12 modules are obtained. Finally, we calculated the correlation between the 18 important immune information screened in the second step and each module divided, as shown in Figure 5.

After calculation, according to the Fisher algorithm described in the method, 1046 immune-related DEGs significantly related to important immune cells screened in the second step are mapped to each WGCNA module. The results are shown in Table 2. The results showed that the DEGs significantly related to important immune cells were significantly enriched in the six modules: black, blue, magenta, purple, red, and yellow, including 68, 289, 96, 54, 117, and 62 immune-related DEGs, respectively.

### 3.4. Construction of the KEGG Cross talk Network Related to Important Genes

**Figure 6:**
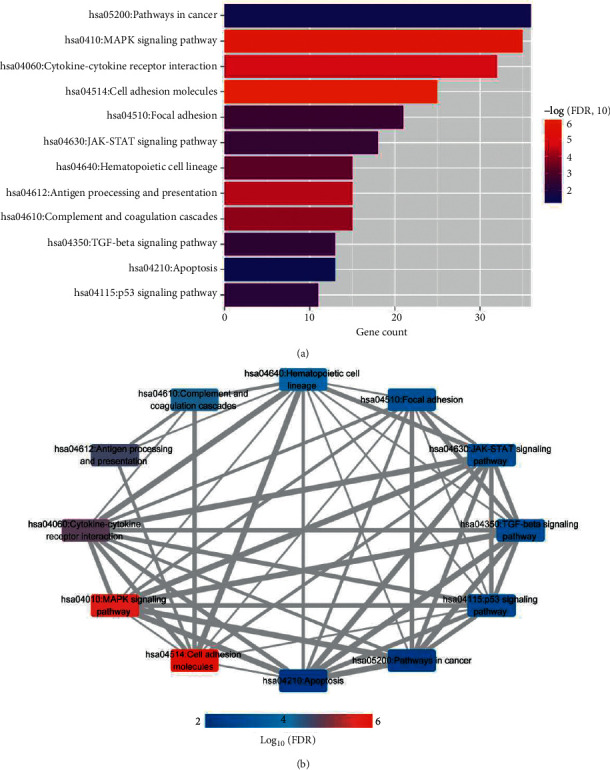
Through KEGG signal pathway enrichment analysis of 686 genes significantly enriched in 6 modules, a total of 12 significantly related frontal KEGG signal pathways were obtained, as shown in Figure 6(a). Then, the quantitative analysis of 12 KEGG signal pathways with significant enrichment correlation was carried out, and 50 pairs of interconnected pairs were obtained after retaining the connection pairs with P value less than 0.05 and absolute value of correlation p value higher than 0.5, so as to construct the cross talk network of KEGG signal pathway, as shown in Figure 6(b).

Through KEGG signal pathway enrichment analysis of 686 genes significantly enriched in 6 modules, a total of 12 significantly related frontal KEGG signal pathways were obtained, as shown in Figure 6(a), and the data information is shown in Table 3. Then, the quantitative analysis of 12 KEGG signal pathways with significant enrichment correlation was carried out, and 50 pairs of interconnected pairs were obtained after retaining the connection pairs with a *p* value less than 0.05 and an absolute value of a correlation *p* value higher than 0.5 so as to construct the cross talk network of the KEGG signal pathway, as shown in Figure 6(b). We will take the immune-related genes involved in 12 internally related KEGG signaling pathways (146 in total) as the follow-up analysis object.

### 3.5. Optimization of Immune-Related Marker Screening and Construction of the Diagnostic Model

**Figure 7:**
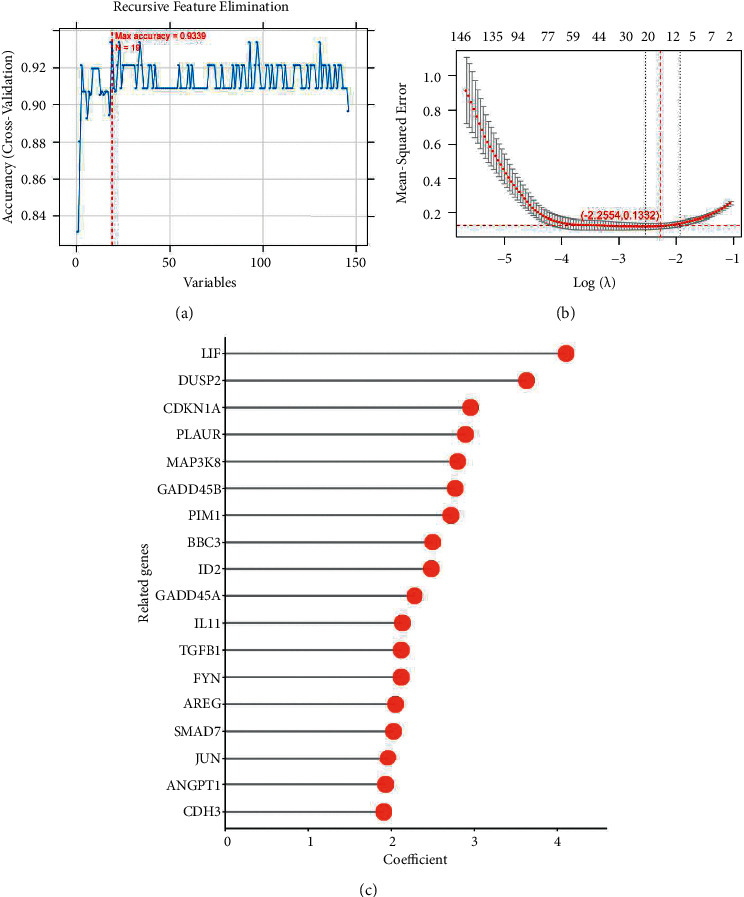
We screened important immune related markers by optimization algorithm. Based on the expression level of 146 DEGs participating in KEGG cross talk network screened in the previous step in the combined data set,Lasso, RFE and RF algorithms are used to screen the optimal combination of DEGs respectively. The parameter diagram of algorithm screening is shown in Figure 7.

We screened important immune-related markers with an optimization algorithm. Based on the expression level of 146 DEGs participating in the KEGG cross talk network screened in the previous step in the combined dataset, LASSO, RFE, and RF algorithms are used to screen the optimal combination of DEGs, respectively. The parameter diagram of the algorithm screening is shown in Figure 7. In LASSO, RFE, and RF algorithms, we screened 15, 19, and 18 DEGs, respectively. By comparing these three sets of DEGs, we got a total of 8 overlapping DEGs. We took 8 DEGs as the final optimal combination of DEGs: AREG, BBC3, DUSP2, map3k8, Smad7, CDKN1A, JUN, and LIF.

An RFE algorithm based on the support vector machine (SVM) is a sequence backward selection algorithm based on the maximum interval principle of SVM. It trains samples through the model, sorts the scores of each feature, removes the features with the minimum feature score, and then trains the model again with the remaining features for the next iteration. Finally, the required characteristic factors are selected. We select the following parameter: cross: 100-fold cross-validation. We select the result with the highest accuracy in cross-validation as the optimal feature diagnosis gene combination under this algorithm. As shown in Figure 7(a), the number of factors with the highest accuracy is 19, and the accuracy is 0.9339.

LASSO regression is a model that adds the constraint term of the L1 norm to the cost function of a linear regression model. It carries out variable screening and complexity adjustment through the control parameter lambda, which is widely used in the field of medicine. Cross-validation fitting then selects the model and has a more accurate estimation of the performance of the model. At this time, the minimum MSE value will be generated, that is, where the red-dotted line crosses as shown in Figure 7(b). At this time, the minimum ordinate MSE value of 0.1332 will be obtained (at this time, the abscissa log (lambda) value is −2.2554), and the nonzero parameter factor under this model is the optimal gene combination under this algorithm.

### 3.6. Construction and Verification of the SVM Diagnostic Model

For the eight characteristic genes screened, we used the decision line method to observe each gene in the training and validation datasets.

**Figure 8:**
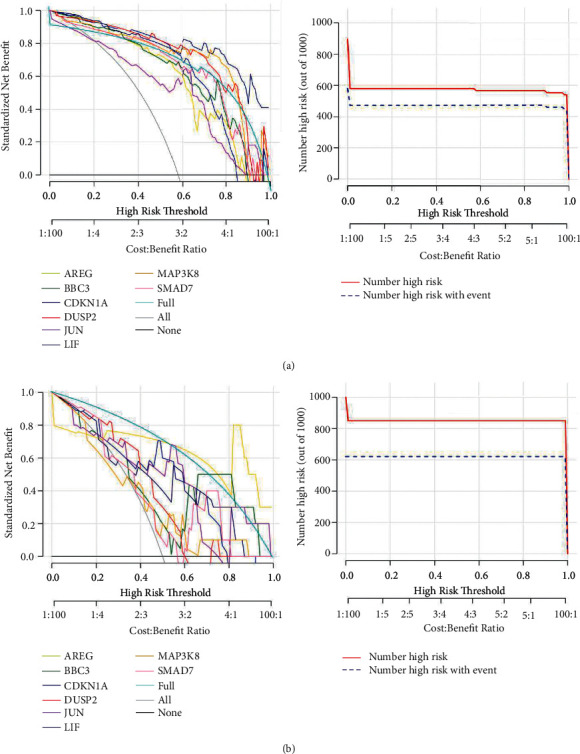
The decisive role in sample determination, and the results are shown in Figure 8.

**Figure 9:**
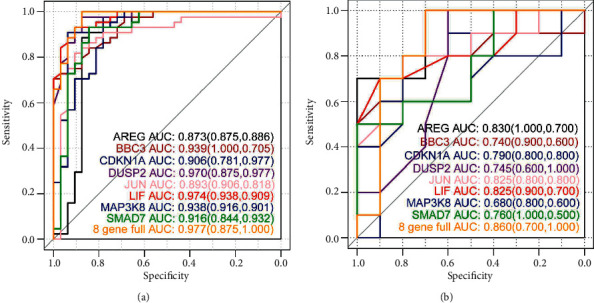
After the classifier is trained and the ROC curve is constructed, as shown in Figure 9.

The decisive role in sample determination and the results are shown in Figure 8. After the classifier is trained and the ROC curve is constructed, as shown in Figure 9, the algorithm is verified based on the SVM training data.

**Figure 10:**
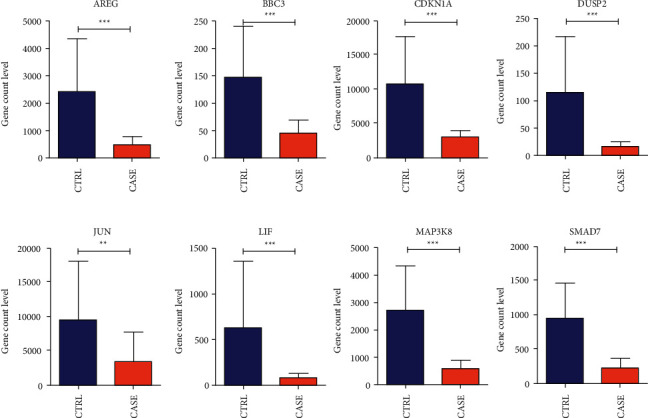
We visually displayed the expression levels of 8 genes in the combined sample, as shown in Figure 10.

**Figure 11:**
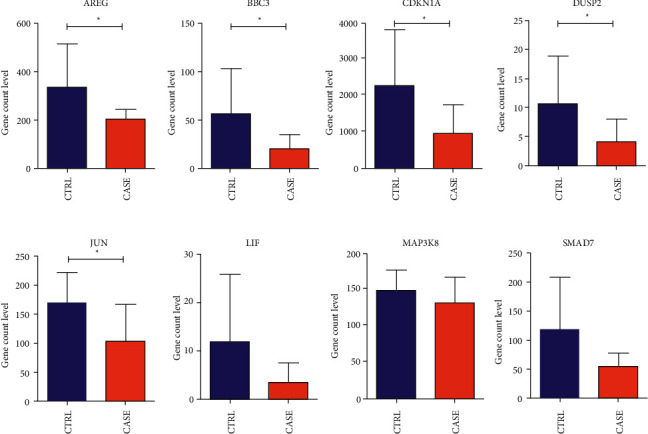
We visually displayed the expression levels of 8 genes in the validation data set GSE112155, as shown in Figure 11.

We visually displayed the expression levels of 8 genes in the combined sample and validation dataset GSE112155, as shown in Figures 10 and 11. The results showed that the expression levels of 8 genes in the GSE112155 validation dataset were completely consistent with the expression differences in the combined training dataset, including AREG, BBC3, CDKN1A, DUSP2, and JUN. The expression levels of the five genes were significantly different between groups.

### 3.7. Expression Level of Characteristic Immune DEGs and Correlation Analysis with Related Immune Cells

**Figure 12:**
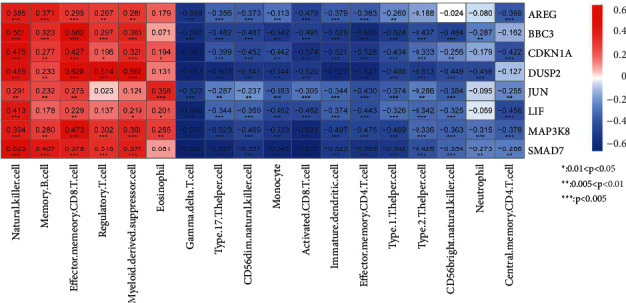
We use R3 6.1 the cor function in the language calculates the PCC between the expression level of 8 characteristic immune DEGs in the combined samples and the 18 immune cell types with significant differences in the distribution between the groups previously screened, and then displays the correlation to observe the correlation between the expression level of characteristic DEGs and related immune cells and the results are shown in Figure 12.

We use R3 6.1 cor function in the language to calculate the PCC between the expression level of 8 characteristic immune DEGs in the combined samples and the 18 immune cell types with significant differences in the distribution between the groups previously screenedand then display the correlation to observe the correlation between the expression level of characteristic DEGs and related immune cells, and the results are shown in Figure 12. The results show that the six immune cells of natural killer cell, memory B cell, effector memory CD8 T cell, regulatory T cell, myeloid-derived suppressor cell, and eosinophil have a high positive correlation with eight characteristic genes and are positively correlated with the remaining 12 immune cells: gamma delta T cell, type 17 T helper cell, and CD56dim natural killer cell. Monocyte, activated CD8 T cell, image dendritic cell, effector memory CD4 T cell, type 1 T helper cell, type 2 T helper cell, CD56bright natural killer cell, neutrophil, and central memory CD4 T cell were negatively correlated with the eight characteristic genes.

## 4. Discussion

Keratoconus is an asymmetric binocular disease [5]. The cornea thins and protrudes in the shape of a lower temporal cone. This corneal deformation will significantly reduce the visual quality. It usually occurs in adolescence and then enters adolescence [5]. Although the etiology is unknown, it is related to genetic factors [6], such as environmental factors [7, 8].

We study the role of immune-related genes through machine learning. The differential expression of KC immune- related genes was obtained. Based on the differential expression of immune-related genes, a KC diagnostic model was established.

Based on the gene expression profile data detected in the combined samples, the immune fine of each sample is calculated by the ssGSEA algorithm. The proportion of 28 immune cell types was obtained. Then, we compare the proportion of various immune cells in kcvs. According to the differences between Ctrl groups, 18 kinds of immune cells with significant differences were obtained: gamma delta T cell, type 17 T helper cell, CD56dim natural killer cell, monocyte, natural killer cell, activated CD8 T cell, image dendritic cell, effector memory CD4 T cell, type 1 T helper cell, memory B cell, effector memory CD8 T cell, regulatory T cell, type 2 T helper cell, CD56bright natural killer cell myeloid-derived suppressor cell, neutrophil, eosinophil, and central memory CD4 T cell.

The combination of KEGG and KEGG-cross parameters in the optimized network is obtained based on the combination of KEGG-cross parameters in the previous step, as shown in Figure 6. In LASSO, RFE, and RF algorithms, we screened 15, 19, and 18 DEGs, respectively. By comparing these three sets of DEGs, we got a total of 8 overlapping DEGs. We took 8 DEGs as the final optimal combination of DEGs: AREG, BBC3, DUSP2, map3k8, Smad7, CDKN1A, JUN, and LIF.

We visually displayed the expression levels of 8 genes in the combined sample and validation dataset GSE112155. The results showed that the expression levels of 8 genes in the GSE112155 validation dataset were completely consistent with the expression differences in the combined training dataset, and the expression levels of AREG, BBC3, CDKN1A, DUSP2, and JUN were significantly different in the comparison between groups.

In this paper, multiple KC-related high-throughput detection data are collected for analysis, and the KC significantly related genes are compared and screened. The important genes significantly related to immunity are screened by using the sample immune evaluation and WGCNA algorithm, and then, the internal correlation of the pathway is analyzed by combining the KEGG cross talk between the important genes. Finally, different optimization algorithms are used to further screen the characteristic genes, and the sample diagnosis classification model is constructed.

Amphiregulin (AREG), one of the ligands of epidermal growth factor receptor (EGFR) 3, has been observed in malignant astrocytoma 4–6 and is responsible for activating complex pathway networks, including Ras/MAPK, PI3K/Akt, PLC *γ*, and stat. The regulation of the listed signal cascade promotes a variety of cellular responses, such as invasiveness, motility, angiogenesis, and proliferation [9, 10]. AREG mRNA consists of six exons and is translated into 252 AA type I transmembrane glycoprotein precursor, pre-AREG. Pro-AREG is expressed on the cell surface, with a hydrophilic extracellular N-terminal, heparin-binding (HB) domain, and EGF-like domain, followed by a parallel membrane handle, including an “exodomain shedding” cutting site (lys187), a hydrophobic transmembrane domain, and a short hydrophilic intracellular cytoplasmic tail [11]. The cleavage of pre-AREG occurs at two N-terminal sites, producing two main soluble forms of AREG (∼19 and ∼21 kDa). In addition, the exodomain shedding of pro-AREG can produce a larger soluble protein of 43 kDa, which is proportional to the whole exodomain. The shedding of the extracellular pre-AREG domain can be initiated by the TACE enzyme, which is a member of the disintegrin and metalloproteinase (Adam) family. After pre-AREG cleavage at lys187, the secretory mature ligand and its blood receptor, epidermal growth factor receptor (EGFR) [4, 9], showed autocrine or paracrine factors on adjacent cells. The interaction between AREG and EGFR triggers a large number of intracellular signal cascades, such as survival PI3K/Akt and mitotic MAPK pathway [9, 12]. There are studies indicating that AREG plays a role in astrocytoma pathogenesis, which indicates the direction for future research of AREG as a tumorigenic factor and promising biomarker in astrocytomas [13]. Our results showed that the expression of AREG in the corneal tissue of KC patients was downregulated compared with the normal control group. This may affect cell mitosis and cause KC.

BBC3 (BCL2-binding component 3), a molecule called p53 upregulated modulator of apoptosis (PUMA), is a member of BCL-2 (B-cell lymphoma 2) family *β* that plays an important role in cell death. The family contains a BCL2-like domain. As an effective activator of apoptosis, BBC3 is expressed in many cells, such as neurons, intestinal, and immune cells and participates in a variety of pathological processes [14, 15]. Recent studies [16] have shown that the BBC3 signal mediates ROS production, DNA damage-dependent cell cycle arrest, and caspase-independent apoptosis in macrophages through the mitochondrial pathway, emphasizing the potential role of BBC3 in the progression of some diseases caused by macrophage dysfunction. Autophagy is an evolutionarily highly conserved process that affects cell development. In the process of autophagy, proteins and organelles in cells are phagocytized, degraded, and recycled by autophagosomes [17]. However, more and more studies [18, 19] show that autophagy is inhibited by tumor promoters and promoted by tumor suppressors in stress cells related to tumor promotion, suggesting that autophagy may be a negative regulator of cell survival. Some studies [20] have shown that BBC3 can further enhance apoptosis by inducing autophagy through Bax activation and mitochondrial outer membrane permeability. In addition, the interaction between autophagy and apoptosis has been widely studied and has been shown to affect many physiological and pathological processes [21, 22]. Autophagy is an important survival-promoting mechanism to maintain metabolic homeostasis under short-term mild stress. However, the apoptotic signaling cascade is initiated after autophagy is overactivated [22, 23]. Our results showed that the expression of BBC3 in corneal tissue of KC patients was downregulated compared with the normal control group. This may affect cell macrophage function and lead to KC.

Cyclin-dependent kinase inhibitor 1a (CDKN1A) is a cell cycle inhibitor that is directly controlled by p53 dependent or independent pathways and participates in terminal differentiation, stem cell renewal, apoptosis, and cell migration.

CDKN1A, also known as p21, encodes an effective cyclin-dependent kinase inhibitor [24, 25]. In developing mouse embryos, CDKN1A expression is associated with cell cycle arrest before terminal differentiation in a variety of tissues [26]. It is well known that overexpression of p21 can induce cell differentiation in a variety of normal and tumor cells, which is mediated by inducing cell cycle regression [27]. p21 functions as a positive or negative regulator of differentiation in a dependent manner [28].

DUSP2, also known as phosphatase that activates cell 1, belongs to a subfamily that mainly plays a role in the nucleus and mainly inactivates ERK.

DUSP2, originally named activated cell phosphatase 1 (PAC-1), is one of the members of bispecific phosphatase (DUSP) and acts as a negative regulator of MAPK through dephosphorylation of phosphotyrosine and phosphoserine/threonine residues [29]. Since DUSP2 was originally found in stimulated human peripheral T cells, most of our current understanding of this phosphatase is about its role in immune response and inflammation [29, 30]. In addition, it has also been suggested that DUSP2 plays a role in apoptosis and cancer [31–33]. The JUN gene, as a downstream transcription factor of the JNK signaling pathway, initiates gene transcription and participates in cell proliferation and differentiation.

Oncogene JUN encodes protein c-JUN, a component AP-1 transcriptional complex that regulates a wide range of series of cellular processes, including cell proliferation and death, survival, and differentiation [34–36].

It is interesting to observe that the expression of amphiregulin, BBC3, cyclin, DUSP2, and JUN in KC is relatively lower than that in the control group. Then, we constructed a nomogram model for diagnosing KC by using amphiregulin, BBC3, cyclin, DUSP2, and JUN. We found that the model has the extraordinary predictive ability, and patients can benefit from the nomogram model at a high-risk threshold of 0 to 1. All these results suggest that abnormal cell proliferation, differentiation, and imbalance of autophagy are one of the most important factors in the pathogenesis of KC. These results may be helpful for the further study of KC. In conclusion, we studied the potential correlation between abnormal cell proliferation and differentiation, imbalance of autophagy, and KC through machine learning and found a close relationship between them. Compared with the control group, some genes related to cell proliferation and autophagy, such as amphiregulin, BBC3, cyclin, DUSP2, and JUN, are underexpressed in KC. Therefore, abnormal cell proliferation and differentiation and an imbalance of autophagy may play an important role in the formation of KC.

## Figures and Tables

**Table 1:** The expression profile data of each dataset before merging, and the data format is the gene read count expression level after standardization under the detection platform of each dataset.

ID	Platform	#Total sample	#Normal	#KC
GSE151631	GPL16791 Illumina HiSeq 2500	26	7	19
GSE77938	GPL18460 Illumina HiSeq 1500	50	25	25
GSE112155	GPL18573 Illumina NextSeq 500	20	10	10

**Table 2:** After batch effect removal, the two datasets tend to be distributed together. The expression spectrum data of the combined datasets are shown in Table 2.

ID	Color	Module size	#Immune related DEGs	Enrichment information	
				Enrichment fold [95%CI]	P_hyper_
Module 1	Black	151	68	2.347 [1.723 – 3.171]	8.15*E* − 08
Module 2	Blue	553	289	2.724 [2.319 – 3.195]	2.20*E* − 16
Module 3	Brown	328	34	0.540 [0.366 – 0.776]	4.62*E* − 04
Module 4	Green	173	—	—	—
Module 5	Yellow green	107	2	0.0975 [0.0116 – 0.361]	2.09*E* − 06
Module 6	Grey	1277	231	0.943 [0.804 – 1.103]	4.82*E* − 01
Module 7	Magenta	131	96	3.819 [2.879 – 5.052]	2.20*E* − 16
Module 8	Pink	147	2	0.0709 [0.00849 – 0.261]	4.23*E* − 09
Module 9	Purple	109	54	2.582 [1.815 – 3.636]	1.49*E* − 07
Module 10	Red	163	117	3.741 [2.897 – 4.819]	2.20*E* − 16
Module 11	Turquoise	2133	91	0.222 [0.177 – 0.277]	2.20*E* − 16
Module 12	Yellow	181	62	1.786 [1.306 – 2.414]	2.70*E* − 04

**Table 3:** The list of DEGs is shown in Table 3.

Term	Count	*p* value	FDR
hsa04514: cell adhesion molecules	25	1.11*E* − 08	6.41*E* − 07
hsa04010: MAPK signaling pathway	35	6.19*E* − 08	1.96*E* − 06
hsa04060: cytokine-cytokine receptor interaction	32	2.02*E* − 06	2.44*E* − 05
hsa04612: antigen processing and presentation	15	3.66*E* − 06	4.01*E* − 05
hsa04610: complement and coagulation cascades	15	1.04*E* − 05	1.09*E* − 04
hsa04640: hematopoietic cell lineage	15	6.10*E* − 05	5.61*E* − 04
hsa04510: focal adhesion	21	2.89*E* − 04	2.37*E* − 03
hsa04630: JAK-STAT signaling pathway	18	4.42*E* − 04	3.28*E* − 03
hsa04350: TGF-beta signaling pathway	13	5.36*E* − 04	3.85*E* − 03
hsa04115: p53 signaling pathway	11	8.85*E* − 04	6.12*E* − 03
hsa05200: pathways in cancer	36	5.14*E* − 03	2.82*E* − 02

## Data Availability

The datasets (GSE151631, GSE77938, and GSE112155) used are available from the comprehensive gene expression database (GEO) at the NCBI GEO (https://www.ncbi.nlm.nih.gov/GEO/) database.
